# Circular RNA hsa_circ_0000658 inhibits osteosarcoma cell proliferation and migration via the miR‐1227/*IRF2* axis

**DOI:** 10.1111/jcmm.16105

**Published:** 2020-12-02

**Authors:** Xin Jiang, Dong Chen

**Affiliations:** ^1^ Department of Orthopedics China‐Japan Friendship Hospital Beijing China

**Keywords:** *circ‐0000658*, circRNA, IRF2, miR‐1227, osteosarcoma

## Abstract

Osteosarcoma (OS) is the most frequently occurring bone cancer. Circular RNAs (circRNAs) have been shown to exert pivotal impact in modulation of gene expression, but their roles in OS are still not fully understood. In this study, we analysed the role of *circ‐0000658* in OS. Thereafter, molecular techniques such as Western blot, qRT‐PCR, RNA‐binding protein immunoprecipitation and Dual‐Luciferase reporter assays were implemented to investigate the role of *circ‐0000658*/miR‐1227/*interferon regulatory factor‐2* (*IRF2*) axis in OS. Eventually, the impact of *circ‐0000658* on tumour growth and metastasis was observed in a xenograft mouse model. The results of this study revealed that *circ‐0000658* exhibits low levels in OS tissues and cell lines. Moreover, *circ‐0000658* repression promoted cell cycle, proliferation, invasion and migration but inhibited the apoptosis of OS cells. Researches have previously shown that *circ‐0000658* contains a binding site for miR‐1227 and thus can abundantly sponge miR‐1227 to up‐regulate the expression of its target gene *IRF2*. Moreover, both inhibition of miR‐1227 and overexpression of *IRF2* reversed cell proliferation and invasion, which was triggered by *circ‐0000658* repression. Conclusively, *circ‐0000658* modulates biological function of OS cells through the miR‐1227/*IRF2* axis. Therefore, *circ‐0000658* might act as a possible novel therapeutic target for the treatment of OS.

## 1 INTRODUCTION

Osteosarcoma (OS) is the most common type of primary bone cancer with malignancy, and it arises from malignant mesenchymal cells producing immature bone and/or osteoid.^1^, ^2^ Moreover, it ranks second in regard to the cause of death associated with cancer in children and teenagers.^3^ Recently, patients with OS exhibiting no distant metastasis have been shown to project a 5‐year survival rate of approximately 65%‐70%, which could be attributed to surgery combined with chemotherapy, a major strategy implemented for treating OS.^4^ However, this rate in patients exhibiting pulmonary metastasis at an early stage is only 20%, in spite of the advances in chemotherapeutics and surgical techniques, as OS exhibits high‐grade malignancy, resistance to chemotherapies and aggressive behaviour.^5^, ^6^, ^7^ Over the last two or three decades, only slight progress has been made in establishing robust treatment methods for OS. Therefore, new effective treatment regimen is urgently required for OS.

Circular RNAs (circRNAs) are a class of non‐coding RNAs (ncRNAs) with limited ability to encode proteins and usually form a closed circular structure covalently joined by the 3′ and 5′ ends.^8^, ^9^ Accumulating evidences about circRNAs have demonstrated that they can impact diverse biological processes and are also implicated in tumour generation and development.^10^, ^11^, ^12^ The most investigated function of circRNAs is as master regulators of gene expression that act to sequester or ‘sponge’ other gene expression regulators, such as miRNAs.^13^, ^14^ For instance, recent studies have indicated that circIFT80 functions as a competing endogenous RNA (ceRNA) of miR‐1236‐3p to promote the development of colorectal cancer.^15^ circFBLIM1 has been shown to act as a ceRNA that promotes the development of hepatocellular cancer by acting as a sponge of miR‐346.^16^ Other studies have confirmed that a variety of circRNAs, such as circRNA_100876,^17^ circRNA_UBAP2,^18^ circ_HIPK3 ^19^ and circRNA_100269,^20^ participate in the pathophysiological processes of OS by exhibiting competitive binding to miRNAs. However, the function of *circ‐0000658* in OS remains unknown.

In this study, we verified that the expression of *circ‐0000658* is low in OS cell lines and tissues. *Circ‐0000658*, as a ceRNA, modulated OS pathogenesis by sponging miR‐1227, thereby promoting the expression of *IRF2* and continuously modulating the behaviour of OS cells. Therefore, *circ‐0000658* might act as a possible novel therapeutic target for the treatment of OS.

## 2 MATERIALS AND METHODS

### 2.1 Collection of OS samples and culture of OS cells

Sixty pairs of OS samples were collected from the patients at the China‐Japan Friendship Hospital. All patients provided the written informed consent, and all assay procedures were conducted based on the approval of the Clinical Research Ethics Committees of China‐Japan Friendship Hospital. The correlation between *circ‐0000658* expression and the clinical and pathological characteristics of patients is presented in Table 1.

**TABLE 1:** Association of *circ‐0000658* expression with clinicopathological features of osteosarcoma

Feathers	Number	High	Low	*P*‐value
All cases	60	30	30	
Age (y)				
<18	41	19	22	0.5796
≥18	19	11	8	
Gender				
Male	26	15	11	0.4348
Female	34	15	19	
Tumour size (cm)				
<5	21	6	15	**0.0292**
≥5	39	24	15	
Histological subtype				
Osteoblastic	5	2	3	0.7076
Chondroblastic	12	6	6	
Fibroblastic	20	12	8	
Mixed	23	10	13	
Distant metastasis				
Absent	25	7	18	**0.0040**
Present	35	23	12	
Clinical stage				
I ~ IIA	24	7	17	**0.0084**
IIB ~ III	36	23	13	

Note

Total data from 60 tumour tissues of osteosarcoma patients were analysed. For the expression of *circ‐0000658* was assayed by qRT‐PCR, the median expression level was used as the cut‐off. Data were analysed by chi‐squared test and Fisher's exact test. *P*‐value in bold indicates statistically significant.

The normal human osteoblast cell line (hFOB1.19) and OS cell lines (HOS, U2OS, SJSA1, Saos2 and MG63) were purchased from Shanghai Cell Bank of Chinese Academy of Sciences (Shanghai, China). The cells were cultured in DMEM (Dulbecco's modified Eagle's medium; Gibco BRL, Grand Island, NY, USA) containing 10% FBS (Foetal bovine serum; Gibco, Carlsbad, CA, USA) in an incubator (Temp: 37°C; CO_2_: 5%).

### 2.2 Real‐time PCR assay, transfection of cells, as well as production and transduction of lentiviruses

TRIzol reagent (Invitrogen) was used for total RNA extraction from OS cells as per the manufacturer's protocol. Thereafter, the extracted total RNA was subjected to reverse transcription to generate cDNA by using the reverse transcription kit from Takara. qPCR was performed using the SYBR Green PCR kit (Takara, Dalian, China). U6 was used for normalization of the miRNA whereas GAPDH was used for the normalization of mRNA or circRNA. The primers used are listed in Table S1.

MiR‐1227, anti‐miR‐1227, miR‐NC, anti‐miR‐NC, *circ‐0000658* shRNA and *circ‐0000658*‐expressing vectors used for cell transfection were synthesized by Ruibo (Guangzhou, China). As per the manufacturer's guidelines, transfection was conducted using lipofectamine 2000 reagent (Invitrogen).

Lentiviral particles carrying scrambled *circ‐0000658* shRNA and *circ‐0000658*‐expressing vectors were generated through HEK293T cells. OS cells were then infected with recombinant lentiviruses, followed by selection with 2 μg/mL puromycin.

### 2.3 Determination of cell proliferation

Cell activity was assessed by using the CCK‐8 Kit (Beyotime, Beijing, China). The transfected cells in each 96‐well plate were cultivated overnight before the addition of CCK‐8 reagent. OD values at 450 nm wavelength were measured by using a microplate reader.

Next, 5‐ethynyl‐2′‐deoxyuridine (EdU) cell proliferation assay was performed by using the Cell‐Light EdU Cell Proliferation/DNA Kit (RiboBio Co., Ltd., Guangzhou, China). Briefly, the cells were immobilized with 4% paraformaldehyde and stained with Apollo Dye Solution, followed by incubation with EdU for 2 hours; finally, the cells were mounted with Hoechst 33342. Thereafter, the images were acquired using a microscope and the number of EdU‐positive cells was quantified.

To assess the colony formation ability of OS cells, monoplast suspension of HOS and MG63 cells was plated in 12‐well plates at the same concentration in each well and incubated in DMEM with 10% FBS. Twelve days later, the colonies visible to the naked eyes were stained, and their images were captured for subsequent counting.

### 2.4 Transwell, cell cycle and apoptosis assays

Cells were inoculated into the transwell chambers that were subjected to 30 minutes of Matrigel coating at 37°C on the upper side, along with 500 μL of complete medium on the bottom side. After 48 hours, the cells on the bottom side were rinsed with PBS, immobilized with 4% paraformaldehyde and stained with crystal violet solution. Eventually, images were captured using a microscope. Analysis of cells in each group was conducted in triplicate.

Thereafter, the cells were trypsinized for separation, rinsed with ice‐cold PBS twice and immobilized with ethanol (70%) at −20°C for overnight. Following day, the cells were suspended in 50 μg/mL of propidium iodide (PI) and 100 μg/mL of RNaseA (KeyGen BioTech: yinghua east street, Chaoyang district, Beijing). The suspended cells were incubated at room temperature for 40 minutes. Eventually, the cells were filtered, and flow cytometry analysis was performed to detect the cell cycle stages.

For apoptosis assay, the cells were rinsed with PBS and stained by using the Annexin V‐FITC Apoptosis Detection Kit (Affymetrix eBioscience: yinghua east street, Chaoyang district, Beijing, China PR) as per the manufacturer's instructions. Then, the FACS flow cytometer (BD Biosciences: yinghua east street, Chaoyang district, Beijing, China PR) was used assess cell apoptosis.

### 2.5 Western blot analysis

RIPA lysis buffer (Thermo Scientific: yinghua east street, Chaoyang district, Beijing, China PR) was used for protein extraction from cells, and the protein content was examined by using the BCA Protein assay kit (Beyotime: yinghua east street, Chaoyang district, Beijing, China PR). Later, electrophoresis was performed for protein separation, and the separated proteins were transferred to a PVDF membrane and blocked with BSA (5%). Thereafter, IRF2‐ (1:1000, Lot No. ab124744, Abcam: yinghua east street, Chaoyang district, Beijing, China PR) and GAPDH‐ (1:500, Lot No. ab9484, Abcam: yinghua east street, Chaoyang district, Beijing, China PR) specific primary antibodies were applied to the membrane, and then, the membrane was incubated overnight at 4°C. Secondary antibodies conjugated with horseradish peroxidase were then used, and the membrane was incubated at room temperature for 1 hour. Finally, the BioSpectrum 600 Imaging System (UVP, CA, USA) was used to obtain the images.

### 2.6 RNase R digestion

The total RNAs (5 μg) were incubated at 37°C for 15 minutes with RNase R (Epicentre Biotechnology, Shanghai, China) that was used to remove the linear RNAs at a concentration of 3 units/1 μg. After RNase R treatment, the expression of *circ‐0000658* was detected *via* qRT‐PCR analysis.

### 2.7 RNA‐binding protein immunoprecipitation experiment

The RNA‐binding protein immunoprecipitation (RIP) assay was performed by using the EZ‐Magna RIP Kit (Millipore, Billerica, MA, USA). After the transfection of miR‐1227 or *circ‐0000658* into the cells, Ago2‐RIP assays were performed. First, the cells were lysed using the RIP lysis buffer along with RNase (Millipore) and proteinase inhibitors (Millipore). Second, the RIP lysates were placed in RIP buffer containing magnetic beads conjugated with human anti‐Ago2 antibody or non‐specific mouse IgG antibody (Millipore). Next, the immunoprecipitates were digested with proteinase K, and the precipitates were examined for *circ‐0000658* expression by RT‐PCR analysis and gel staining. Finally, the RNA concentration was measure by using the NanoDrop spectrophotometer, and qRT‐PCR analysis was conducted by using the purified RNA.

### 2.8 Dual‐Luciferase reporter assay

Following the amplification of 3′‐UTRs of *IRF2* and *circ‐0000658*, they were independently cloned into the firefly luciferase gene downstream in the pGL3 vector (Promega: yinghua east street, Chaoyang district, Beijing, China PR), which were named as wild‐type (WT) 3′‐UTR. According to the manufacturer's instructions, the mutations were induced using the QuickChange site‐directed mutagenesis kit (Stratagene, Cedar Creek, TX, USA), and mutant miR‐1227 binding sites were detected in both *IRF2* and *circ‐0000658* 3′‐UTRs, which were named as MUT 3′‐UTR. OS cells were transfected in combination with WT‐3′‐UTR or MUT‐3′‐UTR and miR‐NC or miR‐1227. After 48 hours of transfection, Dual‐Luciferase reporter assay system (Promega) was used to conduct the luciferase assay. Analysis in each group was performed in triplicate.

### 2.9 Immunohistochemistry

Immunohistochemistry (IHC) was performed as described previously^21^, ^22^ with anti‐*IRF2* antibody using the formalin‐fixed, paraffin‐embedded xenograft tumour tissue sections.

### 2.10 Tumour formation in vivo

Five‐week‐old BALB/c (nu/nu) mice were subcutaneous seeded with 2 × 10^6^ stably transfected HOS cells (*circ‐0000658* or Lv‐NC) in the flank for 4 weeks. Prior to sacrificing the mice, the tumour volume (V) was examined every week, and it was calculated as per the following formula: V = 0.5 × length × width^2^. About 28 days later, cervical dislocation was performed to sacrifice the mice, and the tumours were harvested through surgery and photographed. Thereafter, the tumour tissues were weighed and preserved in liquid nitrogen until further use. As per the US National Institute of Health Guidelines for Use of Experimental Animals, the mice were maintained and experiments were conducted in the SPF Animal Laboratory at the Capital Medical University. The animal experimental procedures were approved by the Ethnic Committee for Experimental Animals of the China‐Japan Friendship Hospital.

Each mouse was inoculated with HOS cells (1 × 10^7^) that were stably injected in the tail vein, so as to establish an advanced‐stage pulmonary metastasis model. Four weeks later, the mice were sacrificed, followed by lung removal, and haematoxylin and eosin (HE) staining.

### 2.11 Statistical analysis

Data are presented as mean ± SD. Differences in categorical variables were determined by using Fisher's exact test, and comparison between the groups was performed by two‐tailed Student's *t* test or one‐way ANOVA. Correlation analysis was performed by assessing Pearson's correlation coefficient. The log‐rank test and Kaplan‐Meier's method were used to assess the survival rates. Differences with *P* < 0.05 were considered as statistically significant.

## 3 RESULTS

### 3.1 *Circ‐0000658* expression level is decreased in OS cells and tissues

**FIGURE 1:**
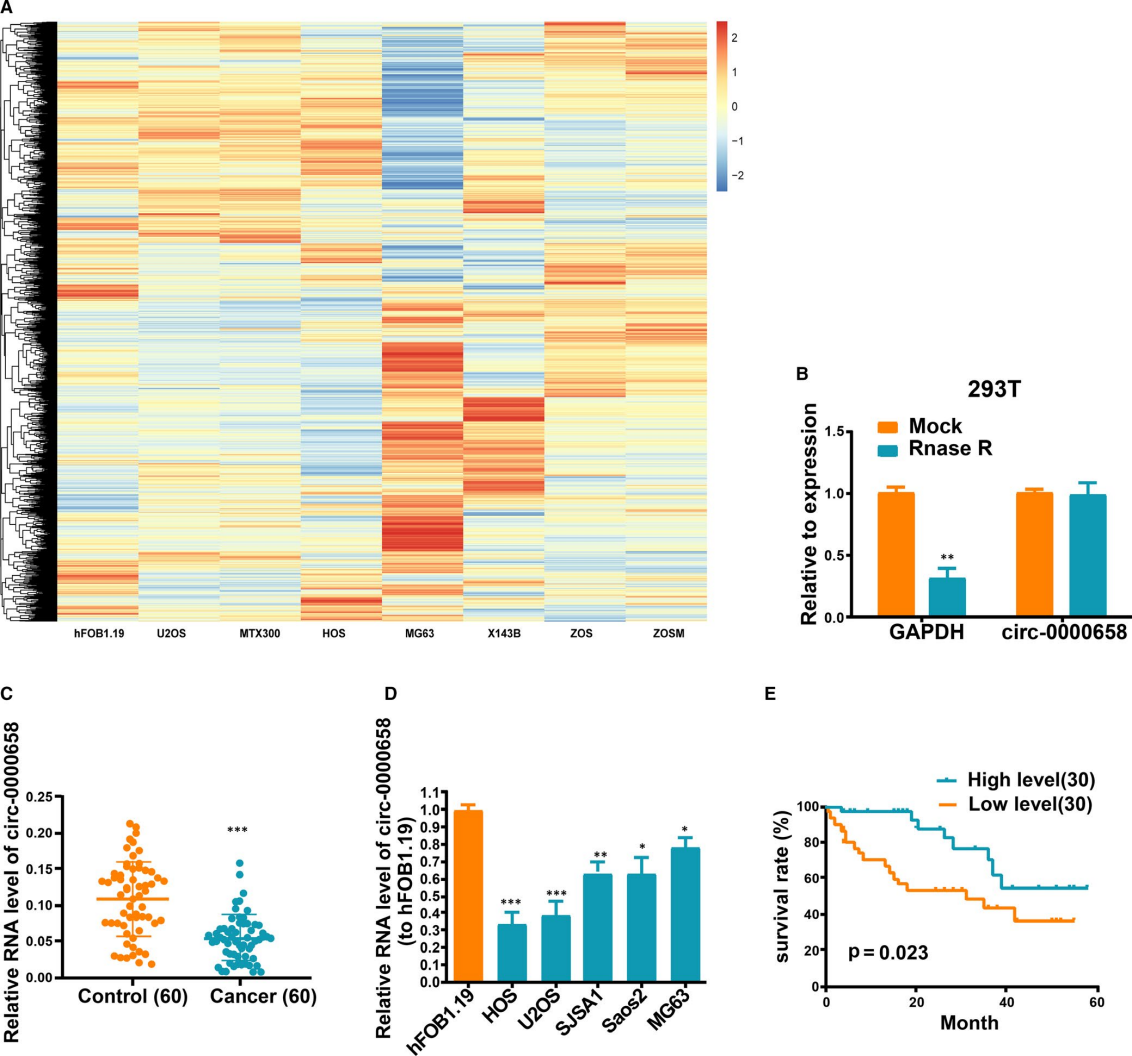
*Circ‐0000658* expression level is reduced in OS tissues and cell lines. The microarray GSE96964 in the platform GPL19978 containing seven human OS cell lines (U2OS, MTX300, HOS, MG63, X143B, ZOS and ZOSM) and the human osteoblast hFOB1.19 was utilized for this analysis. (A) Heatmap of circRNA microarray is presented here. (B) Resistance of *circ‐0000658* in OS cells to RNase R digestion is presented here. (C, D) The expression of *circ‐0000658* in OS tissues and cell lines. (E) High *circ‐0000658* expression level is related to a longer overall survival. Data represent the mean ± SD of 3 independent experiments; *P < 0.05, **P < 0.01, ***P < 0.001

The microarray GSE96964 from platform GPL19978, containing seven human OS cell lines (U2OS, MTX300, HOS, MG63, X143B, ZOS and ZOSM) and the human osteoblast hFOB1.19, was used to perform the expression analysis. Through this analysis, *circ‐0000658*, which is the most significantly down‐regulated circRNA in OS cell lines, was selected as the study subject (Figure 1A). As *circ‐000658* is resistant to RNase R digestion, the circRNA characteristics of *circ‐000658* were corroborated (Figure 1B). qRT‐PCR was performed to examine *circ‐0000658* expression level in the pairs of primary OS tissues and non‐tumour tissues. The results revealed that the non‐tumour tissues expressed *circ‐0000658* at a higher level than the OS tissues (Figure 1C). Similarly, OS cells expressed *circ‐0000658* at a notably lower level than HFOB1.19 cells (Figure 1D).

As presented in Table 1, OS patients were allocated to the high‐expression group (n = 30) and low‐expression group (n = 30), by using the median expression of *circ‐0000658* as the grouping criteria. Reduced *circ‐0000658* expression was not related to gender (*P* = 0.5796), age (*P* = 0.4348) and histological subtype (*P* = 0.7076), but related to distant metastasis (*P* = 0.0040), tumour size (*P* = 0.0292) and clinical stage (*P* = 0.0084). Moreover, Kaplan‐Meier assay revealed that OS patients in high‐expression group exhibit higher overall survival rate than in low‐expression group (Figure 1E).

### 3.2 Circ‐0000658 restricts OS proliferation and invasion in vitro

**FIGURE 2:**
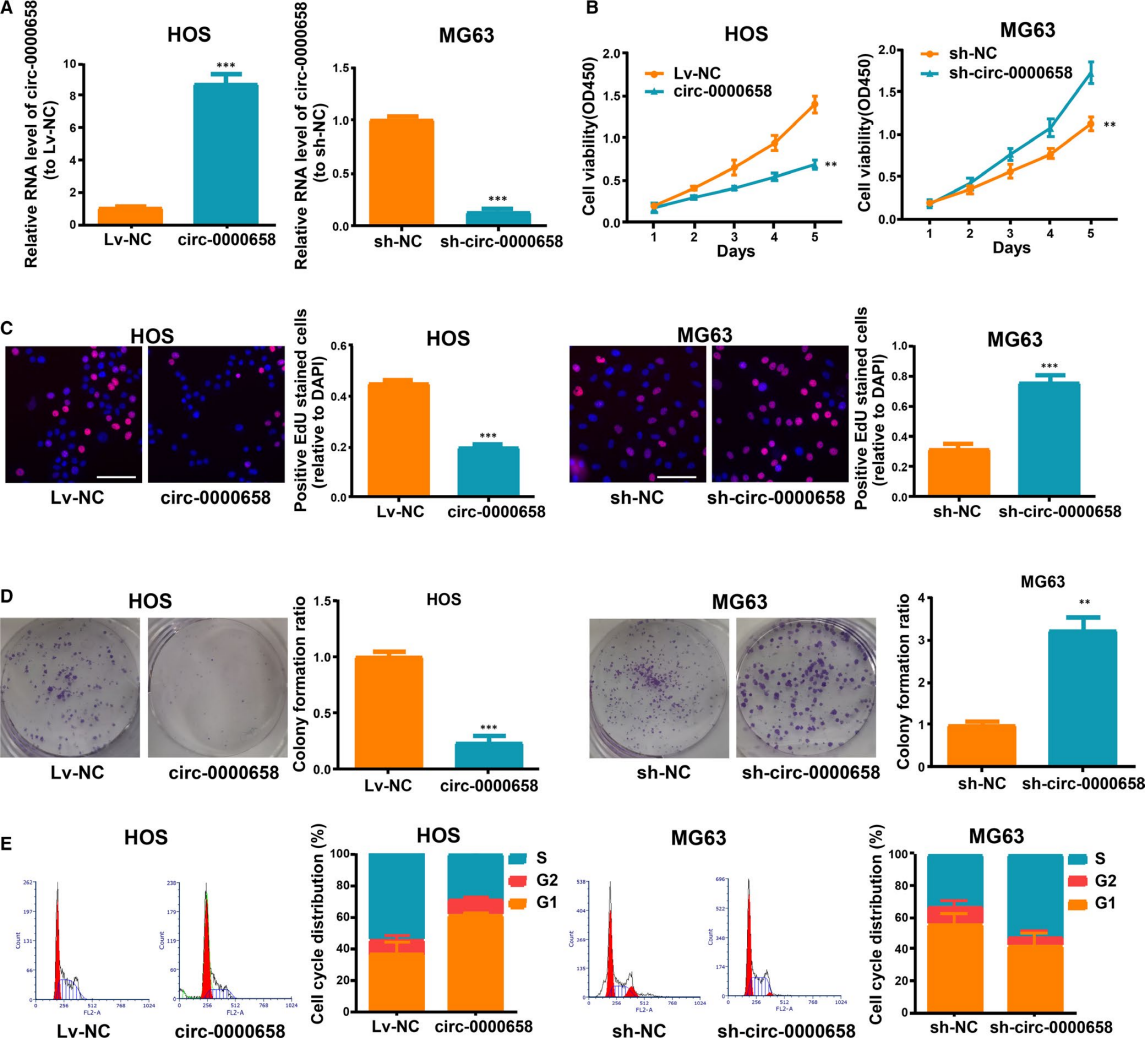
*Circ‐0000658* restricts OS cell proliferation and cell cycle. (A) *Circ‐0000658* expression in MG63 and HOS cells after transfection. (B) CCK‐8 assay, (C) EdU (bar = 100μm) and (D) colony formation assays were performed to figure out the influence of *circ‐0000658* on cell proliferation. (E) The cell cycle of MG63 and HOS cells after transfection. Data represent the mean ± SD of 3 independent experiments; **P < 0.01, ***P < 0.001

**FIGURE 3:**
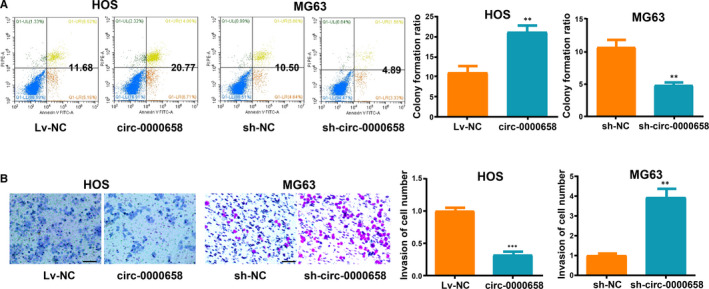
*Circ‐0000658* up‐regulation promotes OS cell apoptosis, but inhibits cell invasion. A, Increase in *circ‐0000658* level aggravates OS cell apoptosis showed by apoptosis assay. B, Increase in *circ‐0000658* level weakens the ability of OS cells to invade showed by transwell assay (bar = 100 μm). Data represent the mean ± SD of 3 independent experiments; **P < 0.01, ***P < 0.001

First, *circ‐0000658* expression level in HOS cells was increased, while it was decreased in MG63 cells after transfection (Figure 2A). Subsequently, it was found that an increase in *circ‐0000658* expression markedly suppressed the cell proliferation and colony formation abilities of cells, as shown in the results of CCK‐8, EdU and colony formation assays (Figure 2B‐D). Moreover, flow cytometry analysis revealed that the S phase in *circ‐0000658* group was lower than in Lv‐NC group (Figure 2E). Later, whether *circ‐0000658* could exert an impact on apoptosis was examined by performing the apoptosis assay. The results revealed that *circ‐0000658* promotes OS cell apoptosis (Figure 3A). Finally, transwell invasion assay was performed to determine the impact of *circ‐0000658* on OS cell invasion. It was found that *circ‐0000658* inhibits OS cell invasion (Figure 3B).

### 3.3 Mutual inhibition between *circ‐0000658* and miR‐1227 expression

**FIGURE 4:**
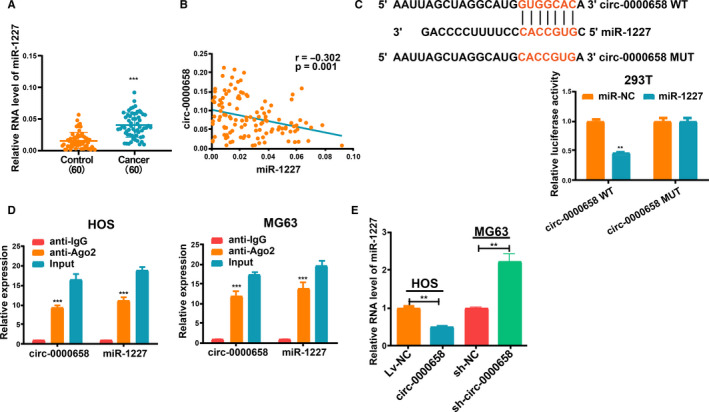
Mutual repression between *circ‐0000658* and miR‐1227. A, qRT‐PCR assay results revealed that the expression level of miR‐1227 is increased in OS tissues. B, Correlation between miR‐1227 and *circ‐0000658* in OS samples. C, The binding sites between miR‐1227 and *circ‐0000658,* and Dual‐Luciferase reporter assay. D, RIP assay in HOS and MG63 cells. E, *Circ‐0000658* inversely modulates miR‐1227 expression. Data represent the mean ± SD of 3 independent experiments; **P < 0.01, ***P < 0.001

MiRNAs with complementary base matching *circ‐0000658* were identified using the CircInteractome (https://circinteractome.nia.nih.gov/); as a result, miR‐1227 was identified, which was previously found to be increased in many cancer cells.^23^, ^24^ qRT‐PCR analysis revealed that miR‐1227 expression was increased in OS tissues than in non‐tumour tissues (Figure 4A) and that there was a negative correlation between the expression levels of *circ‐0000658* and miR‐1227 in OS tissues (Figure 4B). The biding sites of miR‐1227 on *circ‐0000658* are depicted in Figure 4C. The speculated miR‐1227 binding site on *circ‐0000658* (*circ‐0000658* WT) and a mutant miR‐1227 binding site on *circ‐0000658* (*circ‐0000658* MUT) were cloned into reporter plasmids. Co‐transfection with miR‐1227 and *circ‐0000658* WT was shown to markedly weaken the luciferase activity, whereas co‐transfection with miR‐1227 and *circ‐0000658* MUT exerted no such impact on the luciferase activity (Figure 4C). Furthermore, RIP assays verified that *circ‐0000658* and miR‐1227 were gathered in Ago2 immunoprecipitates (Figure 4D). Finally, in both MG63 and HOS cells, sh‐*circ‐0000658* increased and *circ‐0000658* decreased miR‐1227 levels significantly (Figure 4E). Collectively, the above data imply that *circ‐0000658* is able to directly bind to miR‐1227 and thus inversely regulates miR‐1227 expression.

### 3.4 *IRF2* is a direct target of miR‐1227

**FIGURE 5:**
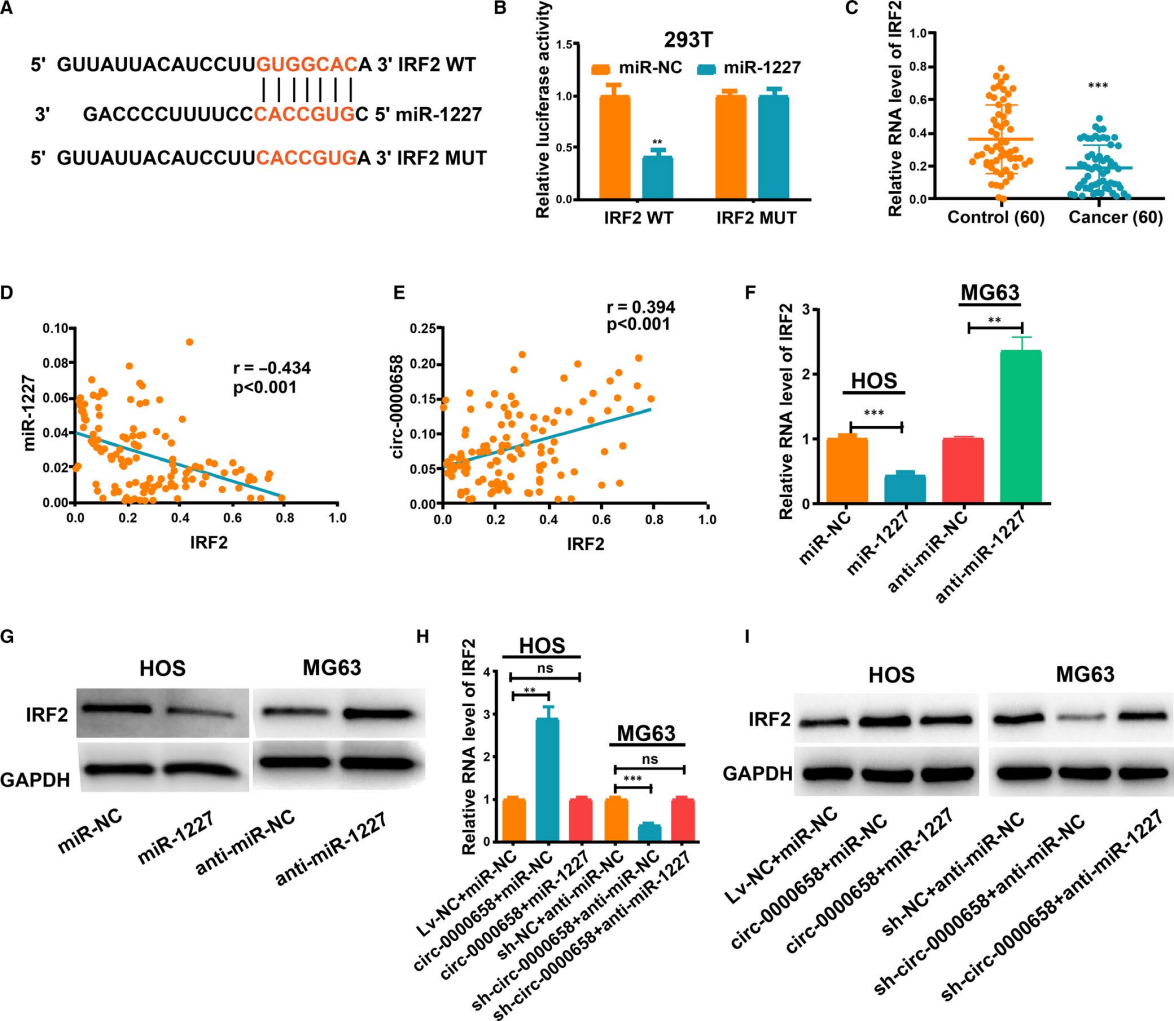
*Circ‐0000658* regulates *IRF2*, which is the target gene of miR‐1227. A, The binding sites between miR‐1227 and *IRF2*. B, Dual‐Luciferase reporter assay in 293T cells. C, *IRF2* expression in OS tissues. D, Correlation between miR‐1227 and *IRF2* in OS samples. E, Correlation between *IRF2* and *circ‐0000658* in OS samples. F, G, qRT‐PCR and Western blot analyses results revealed that miR‐1227 represses *IRF2* expression in OS cells. H, I, Anti‐miR‐1227 treatment reversed the effect of *circ‐0000658* overexpression on *IRF2* expression showed by qRT‐PCR and Western blot assays. Data represent the mean ± SD of 3 independent experiments; **P < 0.01, ***P < 0.001, ns: no statistical significance

*IRF2* was selected from the list of putative target genes of miR‐1227 for future research. The binding sites between miR‐1227 and *IRF2* are presented in Figure 5A. Dual‐Luciferase reporter assay revealed that HEK293T cells co‐transfected with miR‐1227 and *IRF2* WT exhibited reduced luciferase activity relative to other groups (Figure 5B). Then, *IRF2* expression level in OS tissues was evaluated (Figure 5C), and we found that there was a negative correlation between the expression levels of *IRF2* and miR‐1227 in OS, but a positive correlation between the expression levels of *IRF2* and *circ‐0000658* (Figure 5D,E). Moreover, qRT‐PCR and Western blot analyses validated that miR‐1227 inhibited *IRF2* expression in both MG63 and HOS cells (Figure 5F,G). Additionally, the level of *IRF2* was found to be increased upon *circ‐0000658* overexpression, while co‐transfection with miR‐1227 reversed this effect (Figure 5H,I). Altogether, these results suggest that *IRF2* is a downstream target gene of miR‐1227 that can be modulated by *circ‐0000658*.

### 3.5 Increase in *circ‐0000658* expression impedes tumour growth and metastasis

**FIGURE 6:**
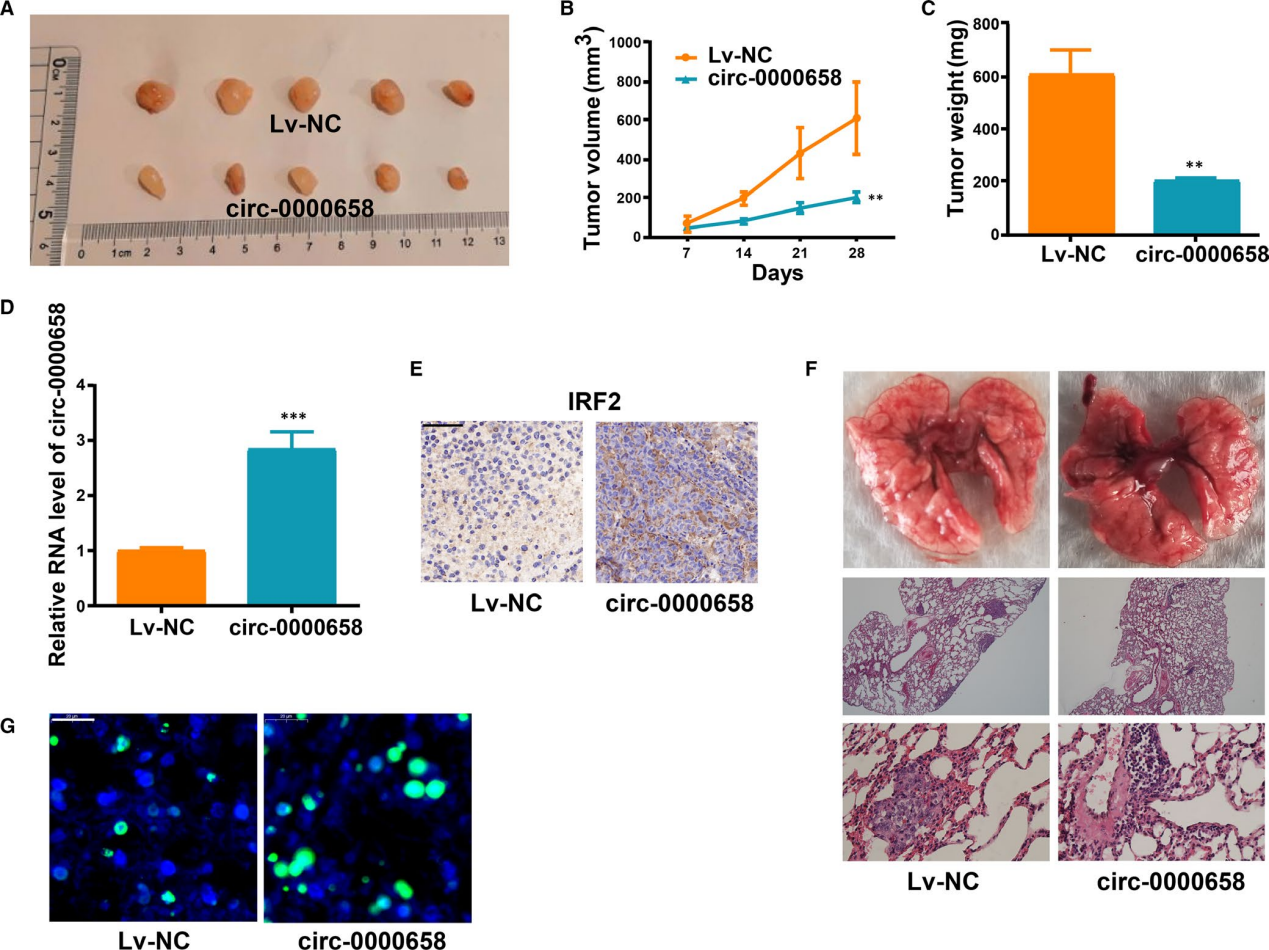
*Circ‐0000658* limits the tumour growth and metastasis in the body. A, Xenograft tumours. B, The growth of xenograft tumours from *circ‐0000658* cells is slower than that of xenograft tumours from Lv‐NC cells. C, The mean weight of xenograft tumours. D, *Circ‐0000658* expression in xenograft tumours was determined. E, *Circ‐0000658* overexpression markedly induces *IRF2* in tumours compared with negative control group (bar = 50 μm). F, Up‐regulation of *circ‐0000658* inhibits tumour metastasis in vivo. Representative macroscopic and microscopic images (H&amp;E staining) of the lungs. G, Up‐regulation of *circ‐0000658* promotes tumour apoptosis (bar = 20 μm). **P < 0.01, ***P < 0.001

Whether an increase in *circ‐0000658* expression impedes tumour growth in the body was investigated further. It was found that the growth of xenograft tumours was reduced upon *circ‐0000658* overexpression (Figure 6A). Moreover, the volume and average weight of xenograft tumours in *circ‐0000658* group were less than those in Lv‐NC group (Figure 6B,C). Thereafter, the expression of *circ‐0000658* in the resected tumour tissues was examined. In addition, upon staining the tumour sections to detect *IRF2* expression, the results revealed that the *IRF2* expression level was also higher in *circ‐0000658* group than in Lv‐NC group (Figure 6E). Further, to investigate whether the increase in *circ‐0000658* expression impedes tumour metastasis, a lung metastasis model was established in vivo. Overexpression of *circ‐0000658* notably reduced lung metastases (Figure 6F). Finally, tunnel staining revealed that the overexpression of *circ‐0000658* notably induced cell apoptosis (Figure 6G).

### 3.6 *Circ‐0000658* regulates OS cell proliferation, invasion, apoptosis and cycle through miR‐1227/*IRF2* axis

**FIGURE 7:**
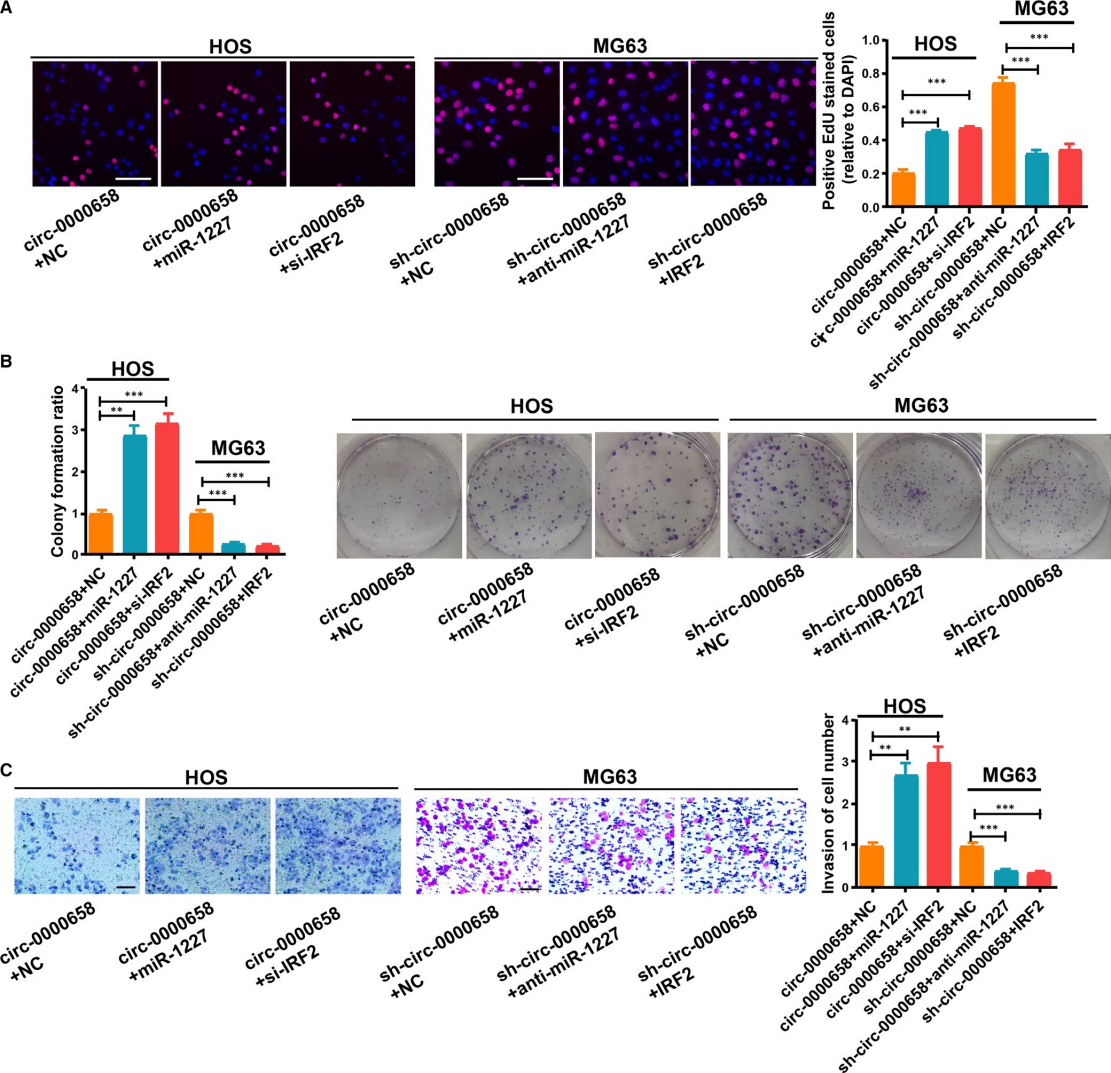
*Circ‐0000658* modulates OS cell proliferation and invasion through miR‐1227/*IRF2* axis. (A) EdU (bar = 100 μm) and (B) colony formation assays were used to assess the cell proliferation of MG63 and HOS cells. (C) Representative images of invasion assay of MG63 and HOS cells (bar = 100 μm). Data represent the mean ± SD of 3 independent experiments; **P < 0.01, ***P < 0.001

To verify the function of *circ‐0000658*/miR‐1227/*IRF2* axis in OS, rescue experiments were conducted on MG63 and HOS cells. HOS cells were transfected with si‐*IRF2* or si‐NC, and MG63 cells were transfected with *IRF2* or vector (Figure S1A,B). Then, EdU, colony formation experiments, transwell invasion, cell cycle and apoptosis assays were performed. The results indicated that both anti‐miR‐1227 and *IRF2* could reverse the impact of sh‐*circ‐0000658* in MG63 cells (Figure 7A‐C, Figure S1C‐F). Additionally, both miR‐1227 and si‐*IRF2* could reverse the impact by *circ‐0000658* in HOS cells (Figure 7A‐C, Figure S1C‐F).

## 4 DISCUSSION

Despite rapid advances in early diagnosis and treatment of OS, most patients develop metastasis and resistance to chemotherapy.^19^, ^25^ It is widely accepted that searching new therapeutic targets and better understanding the pathway related to cancer initiation and progression is essential for improving the prognosis of OS patients. Recently, circRNAs have been demonstrated to exert pivotal effects in the development of different tumours including OS.^26^, ^27^ Thus, circRNA microarray GSE96964 from the platform GPL19978 was used to analyse circRNAs associated with OS, and less‐expressed *circ‐0000658* was selected as the research subject and was validated in cell and tissue populations.

Functional assays revealed that *circ‐0000658* restricted the cell proliferation and invasion and protected cells from undergoing apoptosis to some extent. Moreover, *circ‐0000658* repression was found to promote the G1 to S phase transition of cell cycle. Further, elevated *circ‐0000658* expression group exhibited longer overall survival of OS patients than low *circ‐0000658* expression group. The above‐mentioned findings imply that *circ‐0000658* is a possible biomarker for the prognosis of OS patients and that it inhibits the progression of OS.

The IRF protein family is a pivotal adaptive immune factor and is known to modulate cellular responses implicated in tumour generation.^28^, ^29^ Interferon regulatory factor‐2 (IRF2) of the IRF family is able to exert anti‐oncogenic effects. *IRF2* is down‐regulated in many primary human cancers, including gastric cancer and hepatocellular carcinoma.^28^, ^30^, ^31^ In this study, *IRF2* was found to be expressed in OS tissues at a lower level as compared to that in non‐tumour tissues. Conversely, up‐regulated expression of *IRF2* reversed the promotion of cell proliferation and invasion, which were induced by *circ‐0000658* repression.

Conclusively, the results of this study demonstrate that *circ‐0000658* is notably decreased in OS tissues and can successfully combine with miR‐1227 to regulate *IRF2* expression. Moreover, *circ‐0000658* overexpression inhibits cell proliferation and invasion by targeting the miR‐1227/*IRF2* axis in OS cells. Therefore, *circ‐0000658* may act as a novel therapeutic target for OS treatment and also as a potential biomarker for its prognosis.

## CONFLICTS OF INTEREST

The authors declare that they have no conflicts of interest in this work.

## AUTHOR CONTRIBUTION

**Xin Jiang:** Data curation (equal); Formal analysis (lead); Methodology (lead); Writing‐original draft (lead). **Dong Chen:** Conceptualization (lead); Data curation (equal); Project administration (lead); Supervision (lead).

## Supporting information

Fig S1

Table S1

## Data Availability

The data sets used and/or analysed in the present study can be provided by the corresponding author on reasonable request.
