# Supplementary material for: Circular RNA hsa_circ_0000658 inhibits osteosarcoma cell proliferation and migration via the miR‐1227/IRF2 axis
Source: J Cell Mol Med. 2020 Dec 2;25(1):510–20. doi: 10.1111/jcmm.16105 (PMC7810968; doi:10.1111/jcmm.16105)
Supplement: Supplementary file 1 — Fig S1 [file JCMM-25-510-s001.docx]

**Supplementary Figure 1**

**
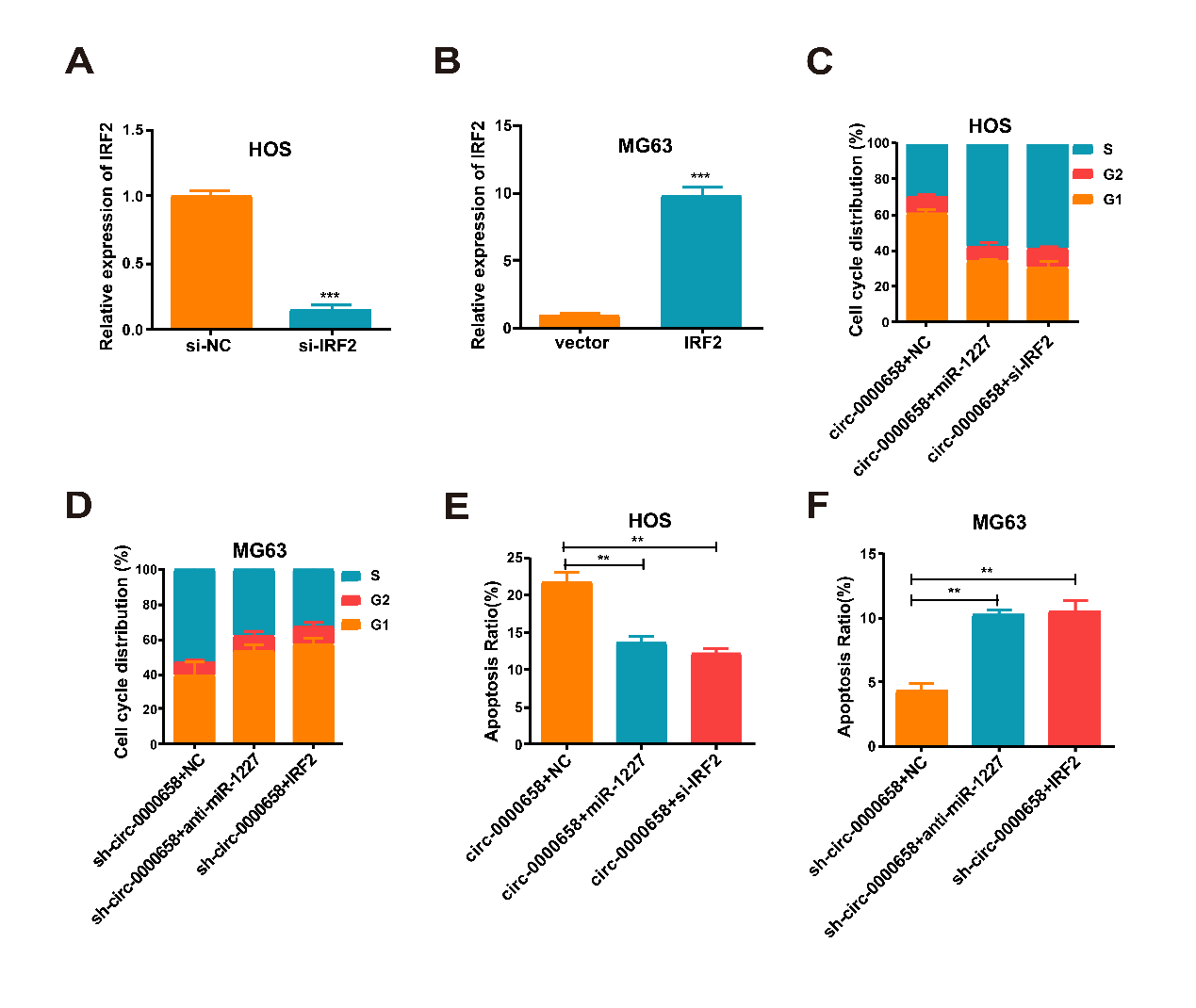
**

**Supplementary Figure 1.** (A) qRT-PCR is used to identify the effect of si-*IRF2* in HOS cells. (B) qRT-PCR is used to identify the effect of *IRF2*-expressing vectors in SW63 cells. (C) Both miR-1227 and si-*IRF2* can reverse the inhibition of *circ-0000658* overexpression on cell cycle. (D) Both anti-miR-1227 and *IRF2* can reverse the influence of sh-*circ-0000658* on cell cycle. (E) Both miR-1227 and si-*IRF2* can reverse the promotion of *circ-0000658* overexpression on cell apoptosis. (F) Both anti-miR-1227 and *IRF2* can reverse the influence of sh-*circ-0000658* on cell apoptosis. Data represent the mean ± SD of 3 independent experiments; ***P*<0.01, ****P*<0.001.
