# Supplementary material for: Circular RNA hsa_circ_0000658 inhibits osteosarcoma cell proliferation and migration via the miR‐1227/IRF2 axis
Source: J Cell Mol Med. 2020 Dec 2;25(1):510–20. doi: 10.1111/jcmm.16105 (PMC7810968; doi:10.1111/jcmm.16105)
Supplement: Supplementary file 2 — Table S1 [file JCMM-25-510-s002.docx]

**Table S1.** **Sequences of primers for qRT-PCR**

| **Name** |  | **Sequence** |
| --- | --- | --- |
| circ-0000658 | Forward | 5’- TTTGGGAGGCTAACATGGGT -3’ |
|  | Reverse | 5’- TCCTGGGTTCAAGCGATTCT -3’ |
| miR-1227 | Forward | 5’- ACACTCCAGCTGGGGACCCCUUUUCCCA -3’ |
|  | Reverse | 5’- CTCAACTGGTGTCGTGGAGTCGGCAATTCAGTTGAGGCACGGT -3’ |
| GAPDH | Forward | 5’- AACGTGTCAGTGGTGGACCTG -3’ |
|  | Reverse | 5’- AGTGGGTGTCGCTGTTGAAGT -3’ |
| U6 | Forward | 5’- CTCGCTTCGGCAGCACA -3’ |
|  | Reverse | 5’- AACGCTTCACGAATTTGCGT -3’ |
| IRF2 | Forward | 5’- CATGCGGCTAGACATGGGTG -3’ |
|  | Reverse | 5’- GCTTTCCTGTATGGATTGCCC -3’ |
